# Acute kidney injury in immunocompromised patients with acute respiratory failure: insights from the HIGH clinical trial and relation with mechanical ventilation

**DOI:** 10.1016/j.aicoj.2026.100048

**Published:** 2026-03-12

**Authors:** Adrien Joseph, Michael Darmon, Laurent Argaud, Kada Klouche, François Barbier, Emmanuel Canet, Guillaume Louis, Alexandre Demoule, Christophe Girault, Samir Jaber, Christine Lebert, Frédéric Pène, Virginie Lemiale, Elie Azoulay

**Affiliations:** a Médecine Intensive et Réanimation, Hôpital Saint–Louis, AP–HP, Paris, France; b Université de Paris, ECSTRA Team, UMR 1153, Center of Epidemiology and Biostatistics, INSERM, Paris, France; c Hospices Civils de Lyon, Hôpital Edouard Herriot, Service de Médecine Intensive–Réanimation, F–69437, Lyon, France, Université de Lyon, Université Claude Bernard Lyon 1, Faculté de Médecine Lyon–Est, F–69373, Lyon, France; d Médecine Intensive et Réanimation, Centre Hospitalier Universitaire de Montpellier, Laboratoire PhyMedExp, INSERM, CNRS, Université de Montpellier, Montpellier, France; e Médecine Intensive Réanimation, Centre Hospitalier Universitaire d'Orléans, 14, Avenue de L'Hôpital, 45100, Orléans, France; f Médecine Intensive Réanimation, Centre Hospitalier Universitaire de Nantes, Université de Nantes, Nantes, France; g Réanimation Polyvalente, CHR Metz–Thionville, 57000, Metz, France; h Médecine Intensive – Réanimation (Département R3S), AP–HP, Groupe Hospitalier Universitaire APHP–Sorbonne Université, Site Pitié–Salpêtrière, INSERM, UMRS1158 Neurophysiologie Respiratoire Expérimentale Et Clinique, Sorbonne Université, Paris, France; i Médecine Intensive – Réanimation, Normandie Univ, GRHVN UR 3830, F–76000 Rouen, France; j Département d'Anesthésie Réanimation B (DAR B), 80 Avenue Augustin Fliche, 34295, Montpellier, France, PhyMedExp, University of Montpellier, INSERM U1046, Montpellier, France; k Médecine Intensive Réanimation, Centre Hospitalier Départemental de Vendée, La Roche–sur–Yon, France; l Médecine Intensive Réanimation, Hôpital Cochin, Assistance Publique–Hôpitaux de Paris, Institut Cochin, INSERM U1016, CNRS UMR8104, Université Paris Cité, Paris, France

**Keywords:** Acute kidney injury, Immunocompromised patients, Mechanical ventilation, Organ cross–talk, Ventilator–induced kidney injury, Acute respiratory failure, Lung–kidney interactions.

## Abstract

**Background:**

Critically ill immunocompromised patients are particularly susceptible to acute kidney injury (AKI) due to various underlying mechanisms. Although invasive mechanical ventilation has been associated with an increased risk of AKI, its specific impact on immunocompromised patients with acute respiratory failure has not been explored. This study aims to describe the prevalence of AKI in this patient population and evaluate the potential risk associated with invasive mechanical ventilation, using causal inference models adjusted for the likelihood of requiring ventilation.

**Results:**

We conducted a post–hoc analysis of 734 immunocompromised patients from the HIGH clinical trial. Of these, 302 (41%) required invasive mechanical ventilation, and 542 (74%) developed AKI. Notably, AKI frequently occurred before the initiation of invasive mechanical ventilation, with the median day of peak KDIGO stage being 2 days (IQR 1–4 days), compared to 3 days (IQR 2–4 days) for initiation of mechanical ventilation. While univariate analysis showed a significant association between invasive mechanical ventilation and AKI (OR = 1.08, 95% CI = 1.02–1.16, p = 0.014), this association was not significant in the multivariate model (OR = 1.05, 95% CI = 0.98–1.13, p = 0.185). Similar findings were observed after adjusting for the risk of invasive mechanical ventilation using overlap weighting and in a competing risk model. Among patients who received mechanical ventilation, 59 (19%) developed AKI after initiation of mechanical ventilation.

**Conclusion:**

Immunocompromised patients with acute respiratory failure face a significant risk of developing AKI, driven by a combination of factors such as their underlying conditions and disease severity. In contrast, the direct impact of invasive mechanical ventilation appears to be limited, suggesting that mechanical ventilation may not be a primary driver of AKI in this vulnerable patient population.

## Background

Lung–kidney interactions have been extensively studied, underscoring the complex and bidirectional relationship between respiratory and renal function in critically ill patients [1]. Patients with acute kidney injury (AKI) are at an increased risk of developing respiratory failure, often necessitating invasive mechanical ventilation [1]. Conversely, a growing body of evidence suggests that acute respiratory failure and the use of invasive mechanical ventilation in itself may exacerbate the risk of AKI [2]. This relationship is particularly concerning in immunocompromised patients, a population that is increasingly represented in intensive care units (ICUs) due to advances in oncology, transplantation, and immunosuppressive therapies. These patients are predisposed to AKI through multiple mechanisms, including direct nephrotoxic effects of treatments, sepsis, and underlying disease processes [3,4].

While invasive mechanical ventilation has been associated with poorer outcomes in immunocompromised patients [5], its specific role in the development of AKI remains unclear.

Understanding this relationship is critical for optimizing the management of these vulnerable patients. This study aims to investigate the prevalence of AKI in immunocompromised patients with acute respiratory failure and to evaluate the potential risks associated with the use of invasive mechanical ventilation in this context.

## Methods

We conducted a post–hoc analysis of the HIGH randomized controlled trial [6], which compared the efficacy of standard oxygen therapy versus high–flow nasal oxygen in immunocompromised patients with hypoxemic acute respiratory failure. The original trial included a diverse cohort of patients, characterized by varying degrees of immunosuppression, defined as use of long–term (>3 months) or high–dose (>0.5 mg/kg/d) steroids, use of other immunosuppressant drugs, solid organ transplantation, solid tumor requiring chemotherapy in the last 5 years, hematologic malignancy regardless of time since diagnosis and received treatments, or primary immune deficiency. Patients with AIDS were not eligible. For this analysis, we included patients who were admitted to the ICU and had sufficient available data on renal function from day 1 to day 28. Patients with pre–existing, dialysis–requiring chronic kidney disease were excluded to focus on incident AKI. AKI was defined and staged according to the Kidney Disease: Improving Global Outcomes (KDIGO) criteria, based on serum creatinine levels and urine output. We monitored AKI progression over the first 28 days of ICU admission, with specific assessments on days 1–7, 14, 21, and 28. Renal recovery was defined as any reduction in peak AKI stage [7]. Chronic kidney disease was defined according to KDIGO guidelines [8].

The primary outcome was the cumulative incidence of AKI, analyzed in the context of death or discharge without AKI as competing risks. The secondary outcomes included the need for renal replacement therapy and the association of invasive mechanical ventilation with the development of AKI. We utilized the Gray test to compare the cumulative incidence of AKI between patients who required invasive mechanical ventilation and those who did not.

To account for potential confounders, we implemented a double adjustment strategy. First, we used overlap weighting, based on a propensity score reflecting the likelihood of receiving invasive mechanical ventilation. This method balances the distribution of baseline characteristics between the two groups, minimizing confounding bias. Variables included in the propensity model were selected a priori based on clinical relevance and previous literature. Secondly, we conducted a multivariate logistic regression analysis, including variables associated with AKI (p < 0.10 in univariate analysis) that were not accounted for in the propensity score model. The Simplified Acute Physiology Score II (SAPSII) was included in the model, excluding the age component to avoid collinearity. Invasive mechanical ventilation was intentionally forced into the final model to assess its independent association with AKI. Categorical data were expressed as numbers and percentages and continuous variables as means and standard deviation (SD). Characteristics of patients with or without AKI were compared using a Student t test or a chi–square test (with continuity correction as appropriate). A sensitivity analysis was conducted to examine the risk of new–onset AKI after mechanical ventilation, either following the initiation of mechanical ventilation or in patients not receiving mechanical ventilation at day 3 (median time of mechanical ventilation initiation).

A two–tailed P value below 0.05 was considered statistically significant. All statistical analyses were performed using R version 4.3.2 (R Foundation for Statistical Computing, Vienna, Austria, https://www.R–project.org/).

## Results

A total of 734 patients from the HIGH trial were included in this analysis. The mean age was 62 years (SD 13), and the baseline serum creatinine level was 87 μmol/L (IQR 71). Of the cohort, 75 patients (10%) had pre–existing chronic kidney disease. The patient population was primarily composed of individuals with onco–hematological diseases (47%), solid tumors (35%), and solid organ transplants (9%) (Table 1) (Supplemental Table S1).

**Table 1:** Baseline characteristics of patients according to acute kidney injury (AKI) incidence during hospitalization.

	All	No AKI	AKI	p
n	734	192	542	
Characteristics before admission				
Age	62.25 (12.56)	60.13 (13.66)	63.00 (12.07)	0.006
Body mass index > 30 kg/m^2^	112 (15.3%)	16 (8.3%)	96 (17.7%)	0.003
Onco–hematological disease (%)	335 (45.6%)	86 (44.8%)	249 (45.9%)	0.834
Solid tumor (%)	260 (35.4%)	75 (39.1%)	185 (34.1%)	0.343
Autologous stem cell transplant (%)	48 (6.5%)	11 (5.7%)	37 (6.8%)	0.398
Allogeneic stem cell transplant (%)	59 (8%)	16 (8.3%)	43 (7.9%)	0.555
Disease status (%)				0.686
Inaugural	253 (34.5%)	70 (36.5%)	183 (33.8%)	
Complete remission	92 (12.5%)	24 (12.5%)	68 (12.5%)	
Partial remission	44 (6%)	11 (5.7%)	33 (6.1%)	
Relapse	128 (17.4%)	37 (19.3%)	91 (16.8%)	
Refractory	55 (7.5%)	16 (8.3%)	39 (7.2%)	
Immunosuppressive drugs (%)	245 (33.4%)	55 (28.6%)	190 (35.1%)	0.231
Corticosteroids (%)	160 (21.8%)	40 (20.8%)	120 (22.1%)	0.230
Solid organ transplant (%)	68 (9.3%)	9 (4.7%)	59 (10.9%)	0.034
Primitive immunodeficiency (%)	2 (0.3%)	1 (0.5%)	1 (0.2%)	0.623
HIV infection (%)	25 (3.4%)	6 (3.1%)	19 (3.5%)	0.991
Type of admission (%)				0.026
Planned surgery	11 (1.5%)	5 (2.6%)	6 (1.1%)	
Unplanned surgery	4 (0.5%)	3 (1.6%)	1 (0.2%)	
Medical	692 (94.3%)	176 (91.7%)	516 (95.2%)	
Comorbidities				
Cardiovascular comorbidity (%)	380 (51.8%)	87 (45.3%)	293 (54.1%)	0.057
Respiratory comorbidity (%)	235 (32%)	74 (38.5%)	161 (29.7%)	0.023
Liver comorbidity (%)	96 (13.1%)	19 (9.9%)	77 (14.2%)	0.176
Baseline serum creatinine ((mean (SD))	87.11 (71.21)	82.21 (47.28)	88.95 (78.30)	0.264
Chronic kidney disease (%)	76 (10.4%)	14 (7.3%)	62 (11.4%)	1.000
Neurological comorbidity (%)	74 (10.1%)	26 (13.5%)	48 (8.9%)	0.079
Diabetes (%)				0.038
No	575 (78.3%)	162 (84.4%)	413 (76.2%)	
Non insulin requiring	93 (12.7%)	16 (8.3%)	77 (14.2%)	
Insulin requiring	44 (6%)	8 (4.2%)	36 (6.6%)	
Charlson score at admission (mean (SD))	5.43 (2.76)	5.38 (2.58)	5.45 (2.82)	0.753
Performans status at admission > 2 (%)	110	21 (10.9%)	89 (16.4%)	0.090
Respiratory diagnoses (%)				0.413
Bacterial pneumonia (documented)	176 (24%)	45 (23.4%)	131 (24.2%)	
Bacterial pneumonia (undocumented)	98 (13.4%)	25 (13%)	73 (13.5%)	
Infiltrative pneumonia	61 (8.3%)	19 (9.9%)	42 (7.7%)	
Pneumocystosis	52 (7.1%)	10 (5.2%)	42 (7.7%)	
Viral pneumonia	31 (4.2%)	9 (4.7%)	22 (4.1%)	
Pulmonary edema	20 (2.7%)	6 (3.1%)	14 (2.6%)	
Invasive pulmonary aspergillosis	14 (1.9%)	1 (0.5%)	13 (2.4%)	
Pulmonary embolism	13 (1.8%)	3 (1.6%)	10 (1.8%)	
Post–transfusion	10 (1.4%)	4 (2.1%)	6 (1.1%)	
Extra–respiratory	67 (9.1%)	14 (7.3%)	53 (9.8%)	
Other	16 (2.2%)	2 (1%)	14 (2.6%)	
Undetermined	176 (24%)	54 (28.1%)	122 (22.5%)	
Respiratory settings (first day of mechanical ventilation)				
Positive End–Expiratory Pressure (mean (SD))	9.03 (3.48)	10.00 (2.65)	8.92 (3.56)	0.441
Arterial Oxygen Partial Pressure/Fraction of Inspired Oxygen ratio (mean (SD))	179.33 (79.77)	202.43 (100.25)	176.76 (77.73)	0.423
Plateau Pressure (mean (SD))	26.44 (5.51)	24.00 (5.02)	26.96 (5.55)	0.237
Bicarbonates (mean (SD))	23.39 (4.69)	25.29 (3.77)	23.17 (4.76)	0.262
pH (mean (SD))	7.37 (0.12)	7.40 (0.11)	7.36 (0.12)	0.435
Arterial Carbon Dioxide Partial Pressure (mean (SD))	41.49 (9.45)	41.71 (9.23)	41.47 (9.55)	0.948
Tidal Volume (mean (SD))	429.81 (134.74)	450.29 (64.32)	427.50 (140.63)	0.675

Categorical data are expressed as numbers (percentages) and continuous variables as means (standard deviation).

**Fig. 1:**
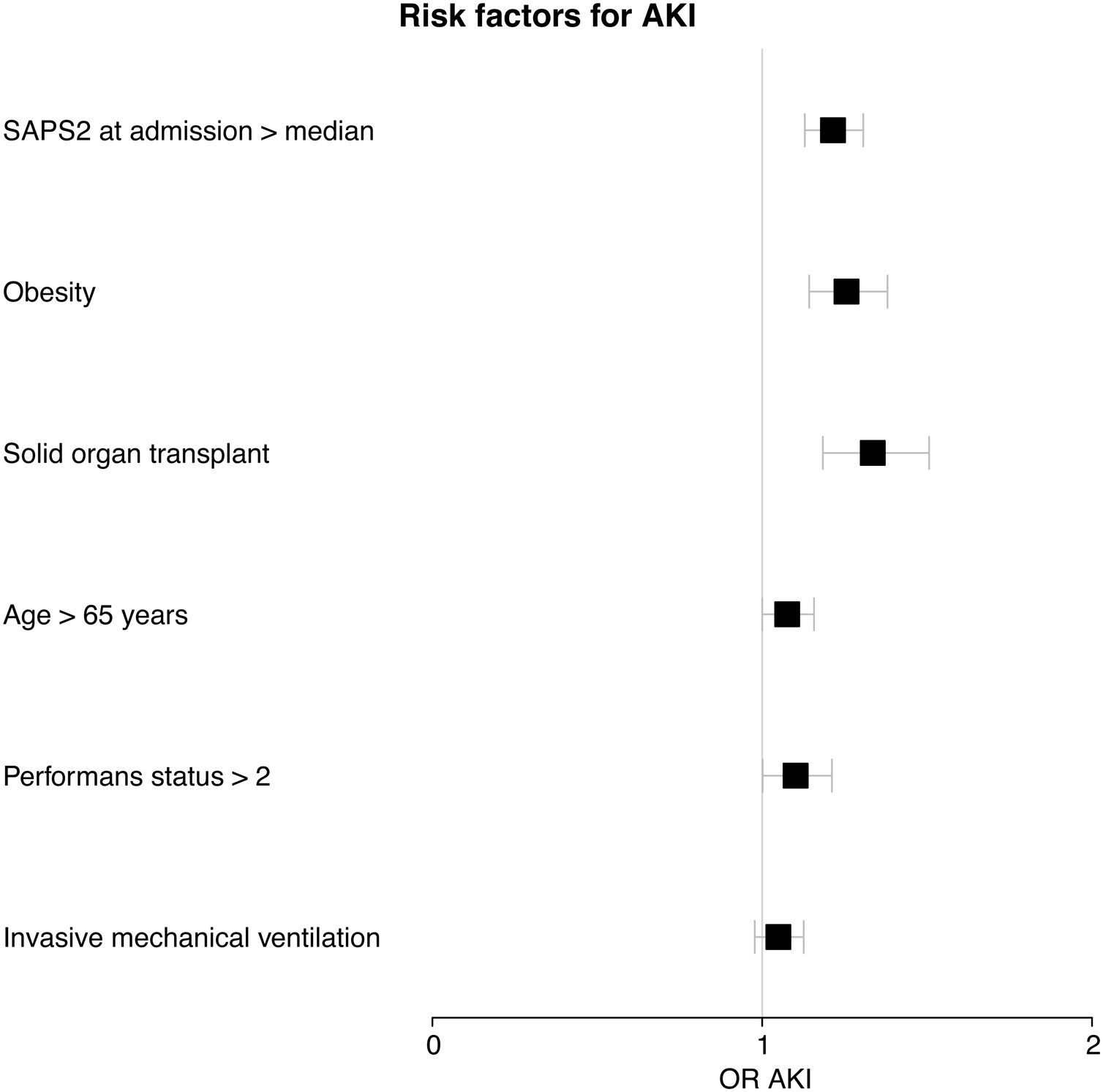
Independent risk factors associated with acute kidney injury in immunocompromised patients with acute respiratory failure. Mechanical ventilation was planned to be forced into the model and was not associated with acute kidney injury (OR = 1.05 [0.98–1.13], p = 0.185).

During their ICU stay, 302 patients (41%) required invasive mechanical ventilation, while 542 patients (74%) developed AKI categorized as stage 1 (n = 53), stage 2 (n = 141), and stage 3 (n = 348), with 138 requiring renal replacement therapy. AKI was defined solely by the urine output criterion in 126 out of 542 patients (23%). Independent risk factors for AKI included obesity, solid organ transplantation, age over 65 years, a SAPSII score above the median, and a Performance Status score greater than 2. During their ICU stay, AKI occurred in 239 out of 302 patients who required mechanical ventilation (79%), 22 out of 33 patients who received only non–invasive ventilation (74%), 167 out of 229 patients treated exclusively with high–flow nasal oxygen (73%) and 161 out of 220 patients treated exclusively with standard oxygen (73%). In univariate analysis, invasive mechanical ventilation was associated with an increased risk of AKI (OR = 1.08, 95% CI = 1.02–1.16, p = 0.014). However, this association was no longer significant after multivariate adjustment (OR = 1.05, 95% CI = 0.98–1.13, p = 0.185) (Fig. 1), except for the most severe forms of AKI (OR for AKI stage 3 = 1.11 [1.03–1.20], p = 0.006)

**Fig. 2:**
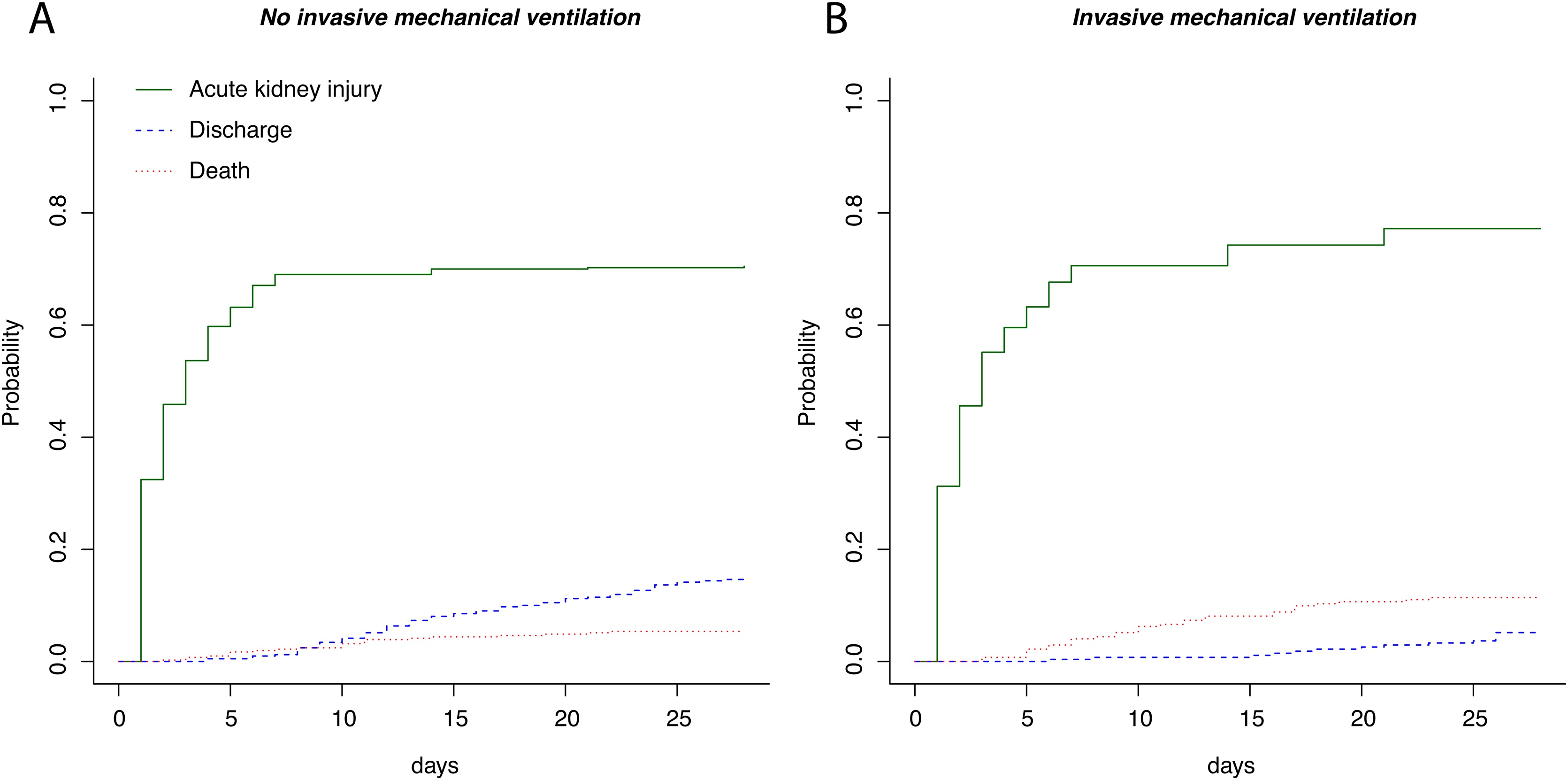
Cumulative incidence of AKI, discharge and death in patients requiring (A) or not (B) mechanical ventilation. Mechanical ventilation was not associated with acute kidney injury in a competing risk model (p = 0.230).

**Fig. 3:**
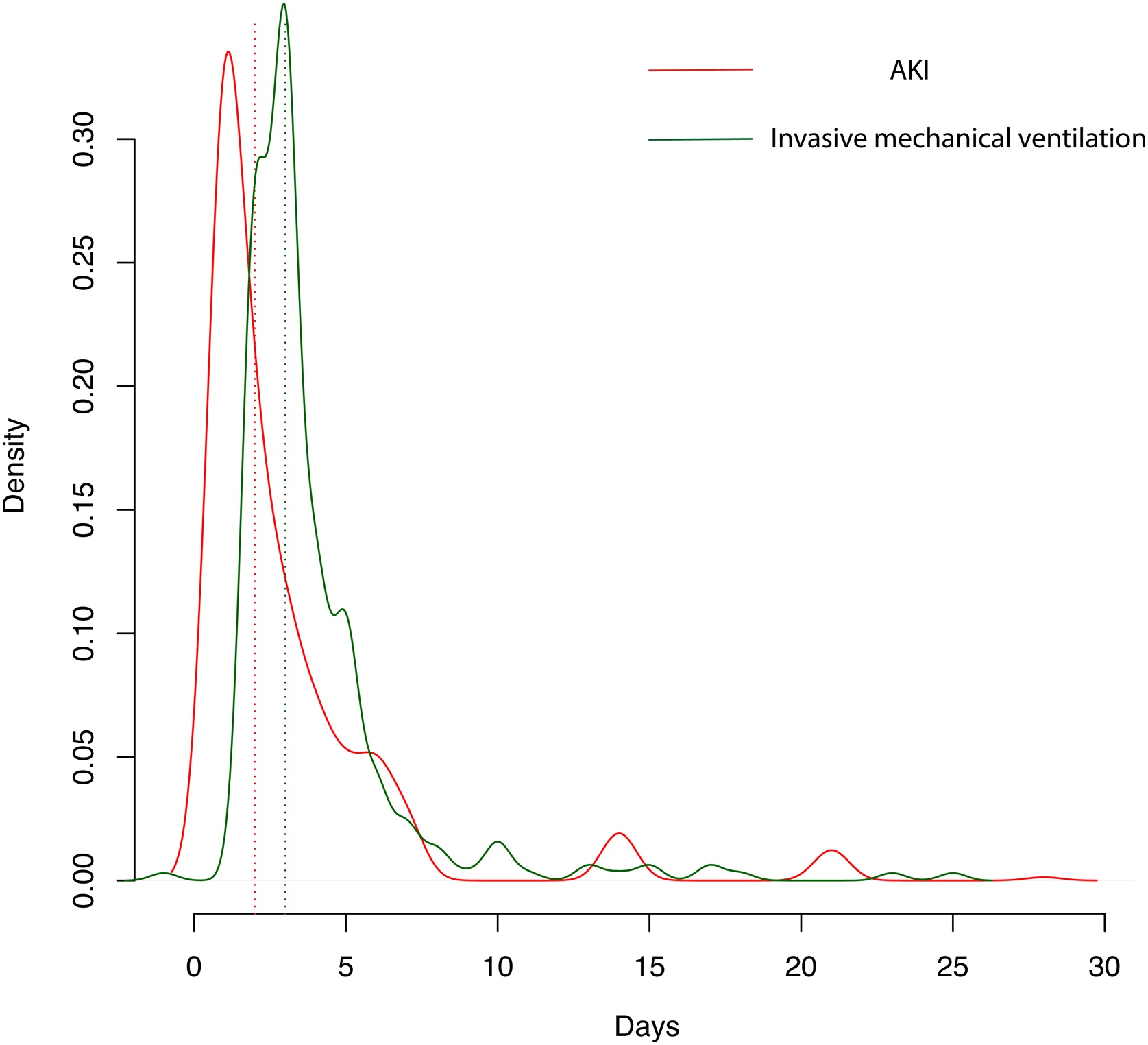
Density curves of acute kidney injury (AKI) and invasive mechanical ventilation after randomization. Median time from randomization to AKI (peak KDIGO stage) = 2 [1–4] days. Median time from randomization to invasive mechanical ventilation is 3 [2–4] days.

Propensity score analysis (Supplemental Fig. S1) and subsequent overlap weighting adjustments confirmed the lack of a significant association between invasive mechanical ventilation and AKI (OR = 1.05, 95% CI = 0.98–1.13, p = 0.143). Moreover, in a competing risk model, the cumulative incidence of AKI did not differ significantly between patients who required invasive mechanical ventilation and those who did not (p = 0.230) (Fig. 2). Interestingly, AKI often preceded the initiation of mechanical ventilation, with a median time to AKI onset of 2 days (IQR 1–4 days), compared to 3 days (IQR 2–4 days) for the start of mechanical ventilation (Fig. 3).

Among the 421 patients who developed AKI prior to mechanical ventilation, 121 (29%) subsequently required intubation. Additionally, 40 patients required RRT before mechanical ventilation, of whom 10 (25%) were later intubated. Of the 302 patients who underwent invasive mechanical ventilation, 59 (20%) developed AKI post–intubation. However, ventilator settings (such as tidal volume and positive end–expiratory pressure) were not associated with an increased risk of AKI (Supplemental Table S2). AKI in these patients was frequently accompanied by multiorgan failure, as indicated by significant increases in SOFA scores across cardiac, respiratory, and neurological domains (Supplemental Fig. S2). Despite the high incidence of AKI, 28–day mortality was 33%, and this outcome was not significantly influenced by the presence of AKI, regardless of whether patients received mechanical ventilation (Supplemental Fig. S3).

A sensitivity analysis focusing on risk of worsening or new onset of AKI after day 3 (patients without mechanical ventilation) or after initiation of invasive mechanical ventilation failed to show any association between invasive mechanical ventilation and AKI (OR = 1.03 [0.97–1.10], p = 0.291).

A separate analysis excluding solid organ transplant recipients produced similar findings: invasive mechanical ventilation was associated with an increased risk of AKI in univariate analysis (OR = 1.08, 95% CI: 1.01–1.16, p = 0.031), but this association was no longer significant after multivariate adjustment (OR = 1.05, 95% CI: 0.98–1.14, p = 0.188).

Among patients with AKI, 180 out of 542 (33%) achieved renal recovery by day 28, while 185 out of 542 (34%) died.

## Discussion

This study underscores the high incidence of AKI among immunocompromised patients with acute respiratory failure, a finding consistent with previous reports in broader ARDS cohorts where AKI rates range between 44% and 62% [[9], [10], [11]]. The even higher AKI incidence observed in our cohort reflects the unique vulnerabilities of immunocompromised patients, who are at heightened risk due to factors such as malignancy–related complications, nephrotoxic treatments, and sepsis [3]. Notably, up to 66% of onco–hematological ICU patients experience AKI, highlighting the critical need for proactive renal monitoring and management in this group [12]. To our knowledge, this study is the first to report AKI incidence in this population, but others have identified immunodeficiency as a risk factor of AKI during acute respiratory failure [7].

Contrary to previous studies that have identified invasive mechanical ventilation as a risk factor for AKI in general ICU populations [2,7] and COVID patients [13] our analysis did not find a significant association between mechanical ventilation and AKI in immunocompromised patients. Several factors may explain this discrepancy. The temporal sequence observed in our study, where AKI often preceded mechanical ventilation, suggests that the development of AKI is more closely related to the underlying disease processes and the severity of the illness rather than the mechanical ventilation itself. Moreover, the lack of association in multivariate analyses, even after adjustment using overlap weighting and in competing risk models, indicates that mechanical ventilation may not be a direct causal factor in AKI development within this population, with the notable exception of AKI stage 3 that remained associated with mechanical ventilation in multivariate analysis. This lack of association may reflect not the absence of MV–related renal effects in immunocompromised patients, but rather the presence of multiple overlapping mechanisms contributing to AKI, which may mask or dilute the specific impact of mechanical ventilation in this complex population. Indeed, the diverse mechanisms of AKI in immunocompromised patients may overshadow any marginal effects of mechanical ventilation. Notably, a recent individual patient data analysis of 5,148 ARDS patients from 10 multicenter randomized controlled trials found no significant difference in AKI incidence across various ventilatory management strategies [11]. The only difference was in the FACTT trial, which compared conservative and liberal fluid management strategies and observed differences in AKI incidence between the control and intervention groups [14,15]. Additionally, non–renal organ failure was significantly associated with increased mortality following AKI. In our cohort, we hypothesize that mortality was predominantly driven by factors such as the severity of the underlying malignancy and overall performance status, which may diminish the independent impact of AKI on outcomes. These findings lead us to suggest that AKI may be more reflective of overall disease severity and that the impact of current standard–of–care mechanical ventilation on AKI might be limited [11,16]. Thus, the precise impact of current mechanical ventilation strategies on the development of AKI remains unclear. Regrettably, several key data points that could provide insights into the mechanisms of AKI in this population were unavailable in our cohort. For instance, information on volume status, nephrotoxic exposure and renal recovery beyond day 28 was lacking. Additionally, concerns have been raised regarding the reliability of the urine output criterion for diagnosing AKI [17,18]. In our study, a substantial portion of the cohort (297 out of 734 patients, 40%) lacked urine output monitoring, which may have impacted our findings. Furthermore, our cohort specifically focuses on immunocompromised patients with acute respiratory failure and may not fully capture the heterogeneity of the immunocompromised population [19].

Our findings suggest that the risk of AKI should not be the primary consideration when deciding whether to initiate invasive mechanical ventilation in immunocompromised patients. Instead, decisions should be guided by the overall clinical picture, including the reversibility of the underlying cause of respiratory failure, the patient's comorbidities, their oncological prognosis, and their personal preferences [20]. Future research should continue to explore the complex interactions between respiratory support strategies and renal outcomes, particularly in vulnerable populations such as immunocompromised patients.

## Conclusion

Immunocompromised patients with acute respiratory failure face a significant risk of developing AKI, predominantly driven by a combination of factors such as their underlying conditions and disease severity. Invasive mechanical ventilation does not appear to independently increase the risk of AKI in this population, suggesting that clinical decisions regarding ventilation should focus on managing the underlying disease processes rather than the potential risk of AKI.

CRediT authorship contribution statement

  • Concept and design: AJ, EA.
  • Acquisition, analysis, or interpretation of data: All authors.
  • Drafting of the manuscript: AJ.
  • Critical revision of the manuscript for important intellectual content: EA, VL, MD.
  • Statistical analysis: AJ, MD.
  • Supervision: EA.

## Ethics approval and consent to participate

The study protocol of the HIGH trial was approved by the CPP Ile de France IV St–Louis ethics committee (March3, 2016, #NIRB00003835/2016/08) and French health authorities (Agence Nationale de Sécurité du Médicament et des Produits de Santé, EudraCT2016–A00220–51). Written informed consent was obtained from all patients or their proxies, in accordance with the Helsinki Declaration of 1975.

## Funding

All financial support for the HIGH study was provided by the French Ministry of Health (P150912 HIGH). No specific funding was obtained for this post–hoc analysis.

## Consent for publication

Not applicable.

## Availability of data and materials

The datasets used and/or analysed during the current study are available from the corresponding author on reasonable request.

## Declaration of competing interest

Dr Azoulay reported receiving travel fees from Gilead and receiving personal fees from Gilead, Astellas, Baxter, Alexion, and Ablynx, not related to the submitted work. Dr Lemiale reported being a member of a research group that has received grants from Fisher & Paykel, Alexion, Baxter, Pfizer, and Gilead, not related to the submitted work. Dr Barbier reported receiving fees from MSD, Pfizer, BioMérieux, not related to the submitted work.

## References

[1] Joannidis M., Forni L.G., Klein S.J., Honore P.M., Kashani K., Ostermann M. (2020). Lung–kidney interactions in critically ill patients: consensus report of the Acute Disease Quality Initiative (ADQI) 21 Workgroup. Intensive Care Med. avr.

[2] van den Akker J.P.C., Egal M., Groeneveld A.B.J. (27 Mai 2013). Invasive mechanical ventilation as a risk factor for acute kidney injury in the critically ill: a systematic review and meta–analysis. Crit Care Lond Engl.

[3] Bridoux F., Cockwell P., Glezerman I., Gutgarts V., Hogan J.J., Jhaveri K.D. (Juin 2021). Kidney injury and disease in patients with haematological malignancies. Nat Rev Nephrol..

[4] Canet E., Zafrani L., Azoulay É (Juin 2016). The critically ill kidney transplant recipient: a narrative review. Chest..

[5] Azoulay E., Pickkers P., Soares M., Perner A., Rello J., Bauer P.R. (Déc 2017). Acute hypoxemic respiratory failure in immunocompromised patients: the Efraim multinational prospective cohort study. Intensive Care Med..

[6] Azoulay E., Lemiale V., Mokart D., Nseir S., Argaud L., Pène F. (27 Nov 2018). Effect of high–flow nasal oxygen vs standard oxygen on 28–day mortality in immunocompromised patients with acute respiratory failure: the HIGH randomized clinical trial. JAMA.

[7] Chawla L.S., Bellomo R., Bihorac A., Goldstein S.L., Siew E.D., on behalf of the Acute Disease Quality Initiative Workgroup 16 (Avr 2017). Acute kidney disease and renal recovery: consensus report of the Acute Disease Quality Initiative (ADQI) 16 workgroup. Nat Rev Nephrol.

[8] Kidney Disease: Improving Global Outcomes (KDIGO) CKD Work Group (Avr 2024). KDIGO 2024 clinical practice guideline for the evaluation and management of chronic kidney disease. Kidney Int.

[9] Darmon M., Clec’h C., Adrie C., Argaud L., Allaouchiche B., Azoulay E. (7 Août 2014). Acute respiratory distress syndrome and risk of AKI among critically ill patients. Clin J Am Soc Nephrol CJASN.

[10] Soto G.J., Frank A.J., Christiani D.C., Gong M.N. (Sept 2012). Body mass index and acute kidney injury in the acute respiratory distress syndrome. Crit Care Med..

[11] Antonucci E., Garcia B., Chen D., Matthay M.A., Liu K.D., Legrand M. (12 Juin 2024). Incidence of acute kidney injury and attributive mortality in acute respiratory distress syndrome randomized trials. Intensive Care Med.

[12] Darmon M., Vincent F., Canet E., Mokart D., Pène F., Kouatchet A. (1 Déc 2015). Acute kidney injury in critically ill patients with haematological malignancies: results of a multicentre cohort study from the Groupe de Recherche en Réanimation Respiratoire en Onco–Hématologie. Nephrol Dial Transplant.

[13] Perschinka F., Mayerhöfer T., Engelbrecht T., Graf A., Zajic P., Metnitz P. (25 Janv 2025). Impact of mechanical ventilation on severe acute kidney injury in critically ill patients with and without COVID–19 – a multicentre propensity matched analysis. Ann Intensive Care.

[14] Liu K.D., Thompson B.T., Ancukiewicz M., Steingrub J.S., Douglas I.S., Matthay M.A. (Déc 2011). Acute kidney injury in patients with acute lung injury: impact of fluid accumulation on classification of acute kidney injury and associated outcomes. Crit Care Med..

[15] (15 Juin 2006). Comparison of Two Fluid–Management Strategies in Acute Lung Injury. N Engl J Med..

[16] Husain–Syed F., Poole D., Joannidis M. (29 Juill 2024). The kidney in acute respiratory distress syndrome: victim or partner in crime?. Intensive Care Med [Internet]..

[17] Machado G.D., Santos L.L., Libório A.B. (12 Août 2024). Redefining urine output thresholds for acute kidney injury criteria in critically Ill patients: a derivation and validation study. Crit Care Lond Engl.

[18] Md Ralib A., Pickering J.W., Shaw G.M., Endre Z.H. (20 Juin 2013). The urine output definition of acute kidney injury is too liberal. Crit Care Lond Engl.

[19] Martinson M.L., Lapham J. (12 Mars 2024). Prevalence of Immunosuppression Among US Adults. JAMA.

[20] Azoulay E., Mokart D., Kouatchet A., Demoule A., Lemiale V. (Févr 2019). Acute respiratory failure in immunocompromised adults. Lancet Respir Med.

