# Supplementary material for: Acute kidney injury in immunocompromised patients with acute respiratory failure: insights from the HIGH clinical trial and relation with mechanical ventilation
Source: Ann Intensive Care. 2026 Mar 12;16:100048. doi: 10.1016/j.aicoj.2026.100048 (PMC12997334; doi:10.1016/j.aicoj.2026.100048)
Supplement: Supplementary file 1 [file mmc1.docx]

**Mechanical ventilation does not seem to increase the risk of acute kidney injury in immunocompromised patients with acute respiratory failure: a post-hoc analysis of the HIGH clinical trial**

*Supplemental material*

Supplemental figure 1: Covariate balance before and after adjustment

Supplemental figure 2: Correlation plot of KDIGO stage and extrarenal organ failures before (A) and after (B) mechanical ventilation

Supplemental figure 3: Survival curves of patients according to acute kidney injury (AKI) and invasive mechanical ventilation (MV)

Supplemental table 1: Characteristics of patients before intensive care unit admission according to acute kidney injury (AKI) incidence during hospitalization

Supplemental table 2: Association between respiratory variables and ventilator settings and risk of worsening renal function after invasive mechanical ventilation


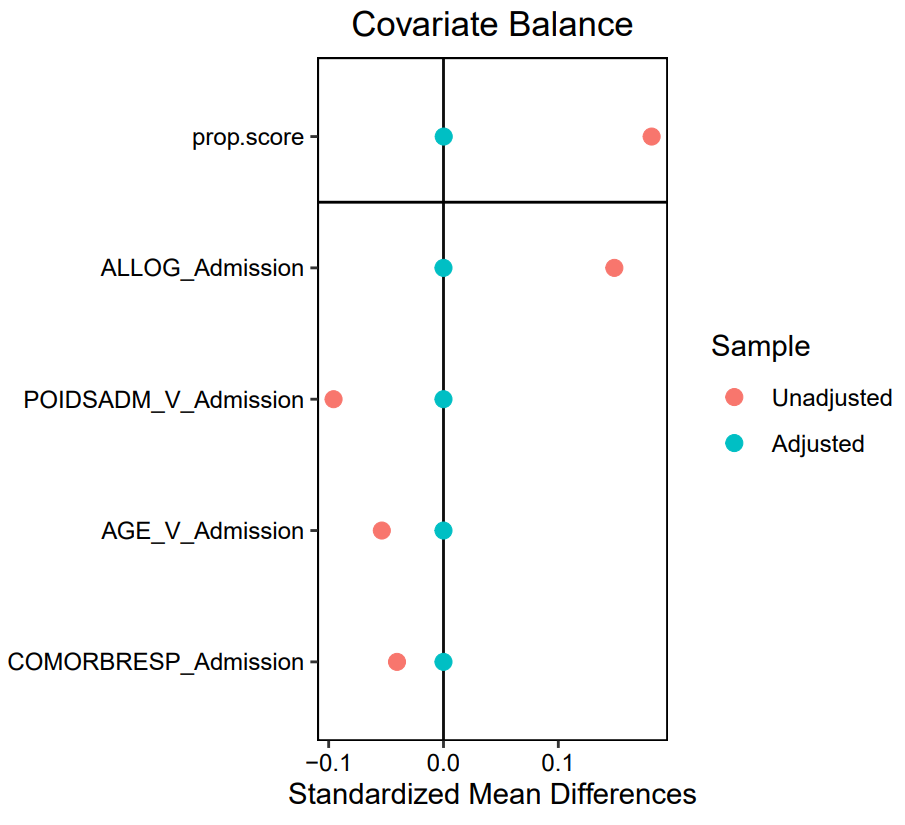


**Supplemental figure 1: Covariate balance before and after adjustment**

| **A** | **B** | |  | |
| --- | --- | --- | --- | --- |
| 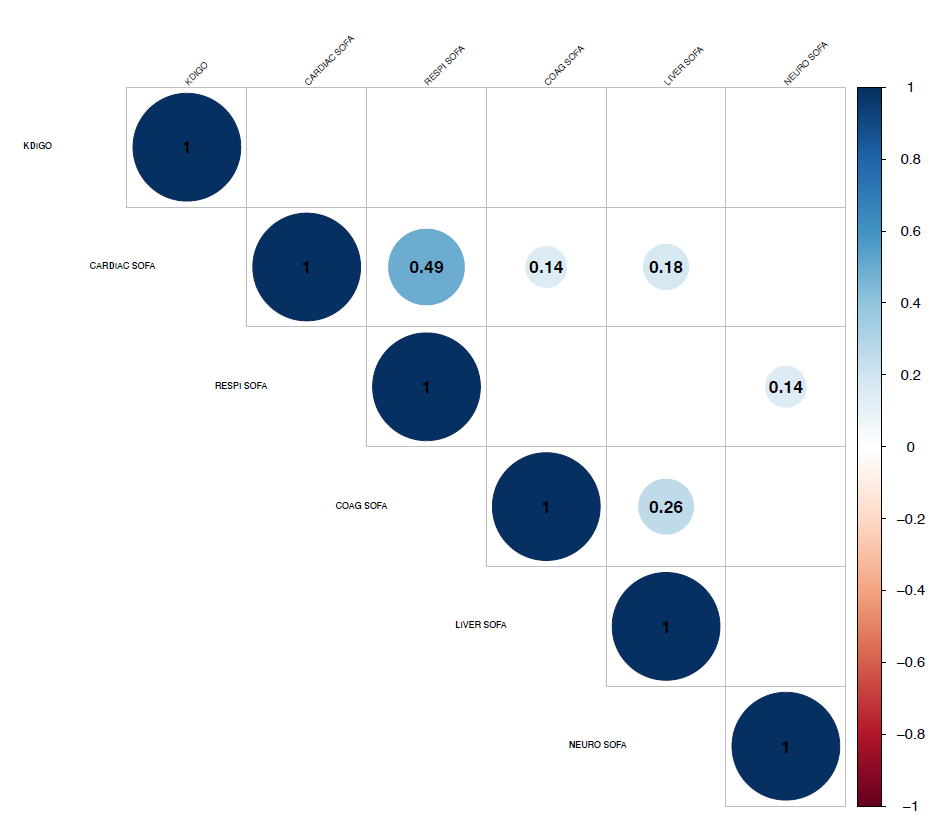 | | 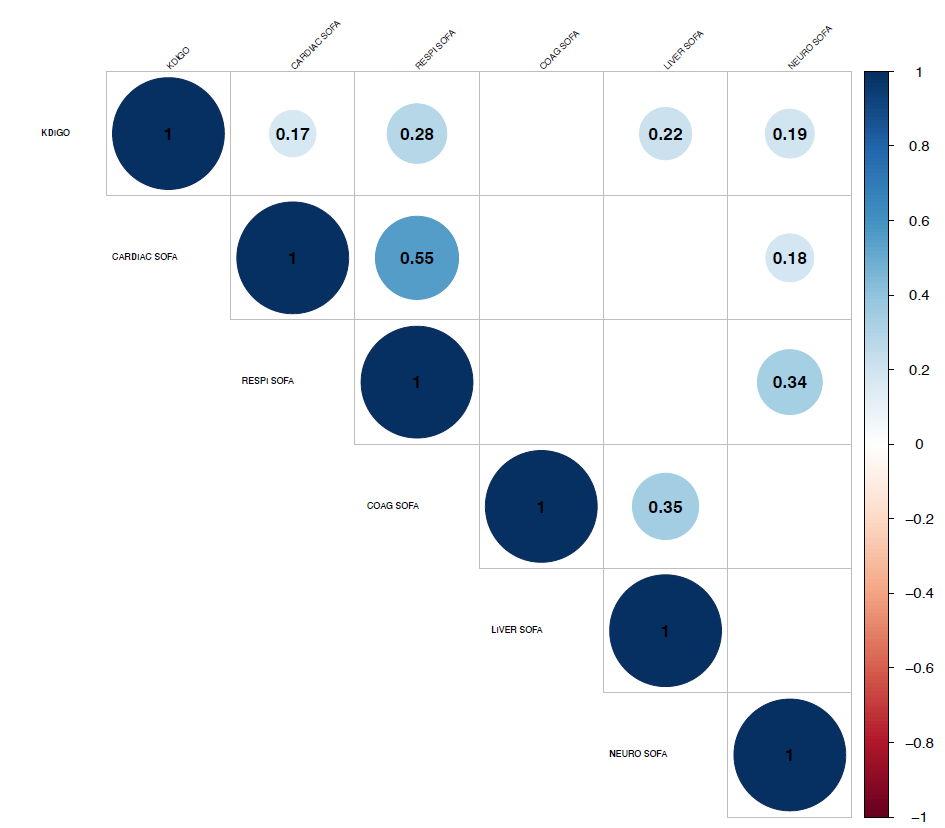 | 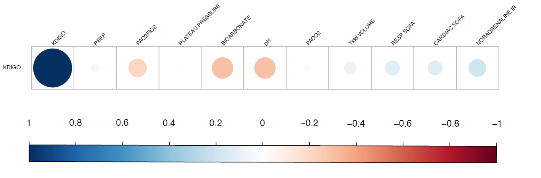 |  |

**Supplemental figure 2: Correlation plot of KDIGO stage and extrarenal organ failures before (A) and after (B) mechanical ventilation**

*The size of the circle is proportional to the significance of the correlation (bigger circles representing lower p values) and the color of the circle represents the correlation coefficient, as displayed in the legend.*


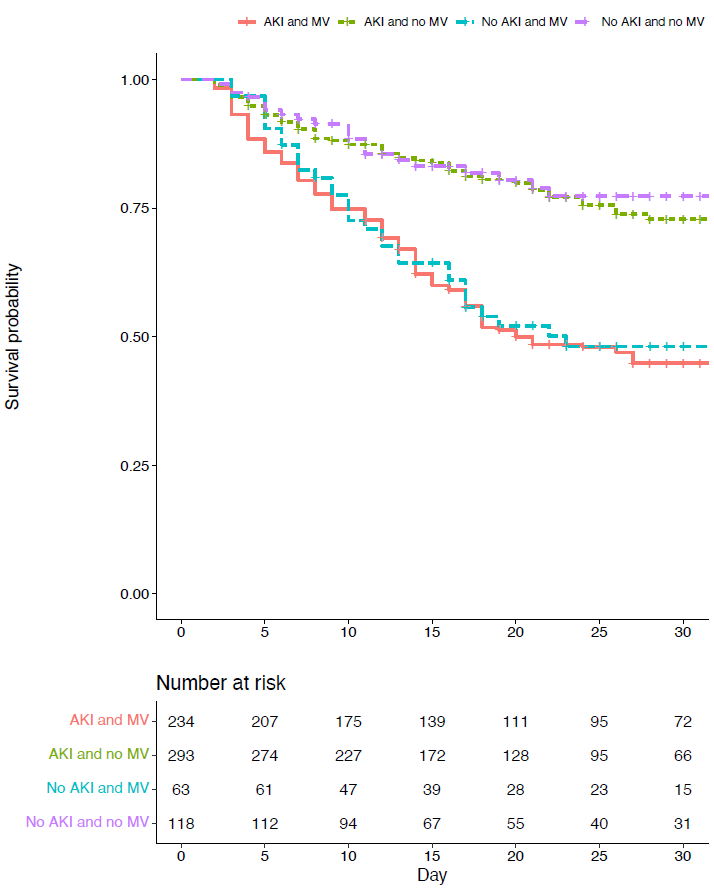


**Supplemental figure 3: Survival curves of patients according to acute kidney injury (AKI) and invasive mechanical ventilation (MV)**

|  | All | No AKI | AKI | p |
| --- | --- | --- | --- | --- |
| n | 734 | 192 | 542 |  |
| **Characteristics before admission** |  |  |  |  |
| Onco-hematological disease (%) |  |  |  | 0.404 |
| Acute myeloid leukemia | 119 (16.2%) | 35 (18.2%) | 84 (15.5%) |  |
| Acute lymphoblastic leukemia | 24 (3.3%) | 8 (4.2%) | 16 (3%) |  |
| Diffuse large B cell lymphoma | 33 (4.5%) | 5 (2.6%) | 28 (5.2%) |  |
| Other agressive B cell lymphoma | 38 (5.2%) | 12 (6.3%) | 26 (4.8%) |  |
| T cell lymphoma | 12 (1.6%) | 6 (3.1%) | 6 (1.1%) |  |
| Waldenström's macroglobulinemia | 2 (0.3%) | 0 (0%) | 2 (0.4%) |  |
| Low grade lymphoma | 12 (1.6%) | 1 (0.5%) | 11 (2%) |  |
| Chronic lymphocytic leukemia | 13 (1.8%) | 2 (1%) | 11 (2%) |  |
| Myelodysplastic syndrome | 21 (2.9%) | 4 (2.1%) | 17 (3.1%) |  |
| Chronic myeloid leukemia | 6 (0.8%) | 2 (1%) | 4 (0.7%) |  |
| Multiple Myeloma | 39 (5.3%) | 6 (3.1%) | 33 (6.1%) |  |
| Other acute hemopathy | 6 (0.8%) | 2 (1%) | 4 (0.7%) |  |
| Other chronic hemopathy | 10 (1.4%) | 3 (1.6%) | 7 (1.3%) |  |
| NA | 1 (0.1%) | 0 (0%) | 1 (0.2%) |  |
| Solid tumor (%) |  |  |  | 0.056 |
| Lung | 71 (9.7%) | 20 (10.4%) | 51 (9.4%) |  |
| Digestive tract | 60 (8.2%) | 13 (6.8%) | 47 (8.7%) |  |
| Breast | 30 (4.1%) | 9 (4.7%) | 21 (3.9%) |  |
| Nasopharyngeal tract | 24 (3.3%) | 12 (6.3%) | 12 (2.2%) |  |
| Urological | 25 (3.4%) | 3 (1.6%) | 22 (4.1%) |  |
| Central nervous system | 6 (0.8%) | 2 (1%) | 4 (0.7%) |  |
| Endometrial | 5 (0.7%) | 1 (0.5%) | 4 (0.7%) |  |
| Ovarian | 1 (0.1%) | 0 (0%) | 1 (0.2%) |  |
| Other gynecologic | 2 (0.3%) | 0 (0%) | 2 (0.4%) |  |
| Testicular | 5 (0.7%) | 4 (2.1%) | 1 (0.2%) |  |
| Bone | 2 (0.3%) | 1 (0.5%) | 1 (0.2%) |  |
| Other | 29 (4%) | 10 (5.2%) | 19 (3.5%) |  |
| NA | 1 (0.1%) | 0 (0%) | 1 (0.2%) |  |
| Disease evolution (%) |  |  |  | 0.366 |
| Localized | 122 (16.6%) | 37 (19.3%) | 85 (15.7%) |  |
| Intermediate | 89 (12.1%) | 27 (14.1%) | 62 (11.4%) |  |
| Diffuse | 296 (40.3%) | 73 (38%) | 223 (41.1%) |  |
| Solid organ transplant (%) |  |  |  | 0.683 |
| Kidney | 40 (5.4%) | 6 (3.1%) | 34 (6.3%) |  |
| Lung | 2 (0.3%) | 0 (0%) | 2 (0.4%) |  |
| Heart | 6 (0.8%) | 2 (1%) | 4 (0.7%) |  |
| Liver | 18 (2.5%) | 1 (0.5%) | 17 (3.1%) |  |
| Kidney/pancreas | 1 (0.1%) | 0 (0%) | 1 (0.2%) |  |
| Other | 1 (0.1%) | 0 (0%) | 1 (0.2%) |  |
| NA | 1 (0.1%) | 0 (0%) | 1 (0.2%) |  |
| Primitive immunodeficiency (%) | 2 (0.3%) | 1 (0.5%) | 1 (0.2%) | 0.623 |
| HIV infection (%) | 25 (3.4%) | 6 (3.1%) | 19 (3.5%) | 0.991 |
| Type of admission (%) |  |  |  | 0.026 |
| Planned surgery | 11 (1.5%) | 5 (2.6%) | 6 (1.1%) |  |
| Unplanned surgery | 4 (0.5%) | 3 (1.6%) | 1 (0.2%) |  |
| Medical | 692 (94.3%) | 176 (91.7%) | 516 (95.2%) |  |
| **Comorbidities** |  |  |  |  |
| Cardiovascular comorbidity (%) | 380 (51.8%) | 87 (45.3%) | 293 (54.1%) | 0.057 |
| Respiratory comorbidity (%) | 235 (32%) | 74 (38.5%) | 161 (29.7%) | 0.023 |
| Liver comorbidity (%) | 96 (13.1%) | 19 (9.9%) | 77 (14.2%) | 0.176 |
| Baseline serum creatinine ((mean (SD)) | 87.11 (71.21) | 82.21 (47.28) | 88.95 (78.30) | 0.264 |
| Chronic kidney disease (%) | 76 (10.4%) | 14 (7.3%) | 62 (11.4%) | 1.000 |
| Neurological comorbidity (%) | 74 (10.1%) | 26 (13.5%) | 48 (8.9%) | 0.079 |
| Diabetes (%) |  |  |  | 0.038 |
| No | 575 (78.3%) | 162 (84.4%) | 413 (76.2%) |  |
| Non insulin requiring | 93 (12.7%) | 16 (8.3%) | 77 (14.2%) |  |
| Insulin requiring | 44 (6%) | 8 (4.2%) | 36 (6.6%) |  |
| Charlson score at admission (mean (SD)) | 5.43 (2.76) | 5.38 (2.58) | 5.45 (2.82) | 0.753 |
| Performans status at admission (%) |  |  |  | 0.286 |
| 0 | 207 (28.2%) | 62 (32.3%) | 145 (26.8%) |  |
| 1 | 199 (27.1%) | 50 (26%) | 149 (27.5%) |  |
| 2 | 159 (21.7%) | 45 (23.4%) | 114 (21%) |  |
| 3 | 83 (11.3%) | 15 (7.8%) | 68 (12.5%) |  |
| 4 | 27 (3.7%) | 6 (3.1%) | 21 (3.9%) |  |
| **Supplemental table 1 : Characteristics of patients before intensive care unit admission according to acute kidney injury (AKI) incidence during hospitalization** | | | | |

|  | **OR [95% CI]** | **p** |
| --- | --- | --- |
| PEEP | 1.000 [0.970-1.032] | 0.976 |
| PaO_2_/FiO_2_ ratio | 0.998 [0.997-1.000] | 0.017 |
| Plateau pressure | 1.023 [0.995-1.052] | 0.124 |
| Bicarbonate level | 1.012 [0.989-1.036] | 0.324 |
| pH | 0.815 [0.323-2.056] | 0.666 |
| P_a_CO2 | 1.002 [0.991-1.014] | 0.694 |
| Tidal volume | 1.000 [0.999-1.000] | 0.950 |
| Respiratory SOFA | 1.042 [1.002-1.083] | 0.040 |
| Cardiac SOFA | 1.028 [0.994-1.062] | 0.107 |
| Noradrenaline infusion rate | 0.995 [0.979-1.010] | 0.504 |
| **Supplemental table 2:** **Association between respiratory variables and ventilator settings and risk of worsening renal function after invasive mechanical ventilation**  *PEEP: positive end-expiratory pressure, SOFA: sequential organ failure assessment.* | | |
